# Correction to “Inhibition of SIRT7 Overcomes Radioresistance in Pancreatic Neuroendocrine Tumors by Reactivating MEN1 Expression”

**DOI:** 10.1002/advs.77330

**Published:** 2026-08-19

J. Jiang, Y. Wang, Y. Qin, et al. “Inhibition of SIRT7 Overcomes Radioresistance in Pancreatic Neuroendocrine Tumors by Reactivating MEN1 Expression.” *Advanced Science* 13, no. 39 (2026): e19824. https://doi.org/10.1002/advs.202519824.

In Section 4.15, **“Establishment and Validation of Radioresistant BON‐1 Cells,”** in the Materials and Methods section, the information regarding the X‐ray irradiator was incomplete.

**The original text**:

“Exponentially growing BON‐1 cells were irradiated using an X‐ray irradiator (SHARP system) at a dose rate of approximately 1 Gy/min.”

**Should have read**:

“Exponentially growing BON‐1 cells were irradiated using an X‐ray irradiator (SHARP 100 system, Raycision Medical Technology Co., Ltd.) at a dose rate of approximately 1 Gy/min.”

This correction provides the complete identification of the irradiation system used in this study to improve methodological transparency and reproducibility. It does not affect the results, data interpretation, or conclusions of the article.

We apologize for this error.

